# Circadian disruption is associated with altered postural control in aged individuals under eye closed condition

**DOI:** 10.3389/fnins.2025.1574544

**Published:** 2025-04-30

**Authors:** Hóngyi Zhào, Zhiqiang Zhang, Yinxia Bai, Peng Li, Yonghua Huang

**Affiliations:** ^1^Sleep Center, NO 984 Hospital of PLA, Beijing, China; ^2^Department of Neurology, The Seventh Medical Center of PLA General Hospital, Beijing, China; ^3^Department of Psychiatry, Inner Mongolia Mental Health Center (The Third Hospital of Inner Mongolia Autonomous Region, Brain Hospital of Inner Mongolia Autonomous Region), Hohhot, China; ^4^Department of Neurology, Mental Health Institute of Inner Mongolia Autonomous Region, The Third Hospital of Inner Mongolia Autonomous Region, Hohhot, China; ^5^Department of Neurology, Shanghai Forth People Hospital Affiliated to Tongji University, Shanghai, China

**Keywords:** actigraphy, circadian rhythm, postural balance, posturography, visual input

## Abstract

**Background:**

Sleep loss is reported to affect postural control. However, the relationship between increased postural sway and the circadian rhythm (CR) remains unclear.

**Objectives:**

To assess performance in the postural control test in aged individuals with an abnormal CR.

**Methods:**

This cross-sectional observational study included two groups of participants: those at high risk of falling (HFR) and those at low risk of falling (LFR), which was determined by the clinical cut-off score for the sway path with open eyes. Each participant wore an ActiGraph device on their non-dominant hand for 5–7 days. A non-parametric analysis of CR variables, including interdaily stability (IS), intraday variability (IV), relative amplitude (RA), interdaily coefficient of variation (ICV), etc., was used to evaluate the postural stability with a posturographic platform during a 30-s static balance test under the eyes closed (EC) and eyes open (EO) condition.

**Results:**

Individuals in the HFR group demonstrated significantly higher scores in the Downton fall risk index (DFRI), higher ICV, and lower IS and M10 activity counts than the LFR group. Linear regression analysis revealed that under the EO condition, there was no association between postural control and CR disruption; however, under the EC condition, L5 was positively associated with variables reflecting an increase in postural sway.

**Conclusion:**

Increased postural sway was found to be associated with CR disruption in aged adults under the EC condition.

## Introduction

Falling is common in geriatric individuals and stands as the second leading cause of unintentional or accidental injury-related deaths globally (Dokuzlar et al., 2020). Aging contributes to a natural decline in postural control due to alterations in the musculoskeletal and neuromuscular systems, which affects complex motor performance and elevates the risk of falls (Jalali et al., 2015; McManus et al., 2022).

An individual’s physiological functions undergo periodic changes, known as the circadian rhythm (CR). The suprachiasmatic nucleus (SCN) in the hypothalamus governs the circadian system and is influenced by signals from internal molecular mechanisms. It functions to regulate nearly all biological processes, including hormone secretion, energy metabolism, body temperature, and sleep–wake function (Musiek et al., 2018; Zhào et al., 2025). The CR is impacted in later life, with a potential reduction in circadian amplitude and increased lability in circadian phases (Goswami et al., 2020; Shim et al., 2024). Furthermore, some of these changes have been linked to a higher risk of chronic diseases and disorders related to mobility and cognition (Kume et al., 2020; Smagula et al., 2020; Feng et al., 2023).

Previous systematic reviews have demonstrated that both sleep loss and circadian arrhythmicity influence postural control (Izadi et al., 2022; Umemura et al., 2022); however, most studies have concentrated on the effects of sleep deprivation, exploring the role of the CR using forced desynchrony (Sargent et al., 2010). The characteristics of the CR extend beyond the scope of prior research. To our knowledge, only one study has attempted to establish a connection between the CR variable L5 (movement activity intensity during the least active 5 h of the day) and postural control performance (Furtado et al., 2016). Given that sleep homeostasis and CR function independently (Umemura et al., 2022), this study aimed to determine whether elderly individuals at a high risk of falling exhibit an abnormal CR pattern. Additionally, we sought to identify the relationship between variables derived from a non-parametric analysis of the CR and postural stability.

## Materials and methods

### Participants

Through two sample *t*-tests using effect size, according to the pilot study launched by Furtado et al. (2016), we assumed the following stability index score under the eyes open (EO) condition: *δ* = μ1 − μ2 = 0.25, *σ* (standard deviation) = 0.35. Group sample sizes of 35 and 35 were required to achieve 80.308% power to reject the null hypothesis of equal means. In the current study, 50 aged adults voluntarily participated in the study. Individuals who scored ≥ 350.63 in the clinical cut-off scores for sway path with EO for 30 s were grouped into the high falling risk (HFR) group (*n* = 25), whereas those who scored <350.63 were grouped into the low falling risk (LFR) group (*n* = 25) (Wiśniowska-Szurlej et al., 2022). The group allocation was blinded to assessors.

This study received approval from the Academic Ethics Committee of the Biological Sciences Division at the NO 984 Hospital in Beijing, China. All participants signed informed consent. The exclusion criteria comprised musculoskeletal, rheumatological, neurological, visual, and vestibular diseases, in addition to habits of alcohol consumption, strong tea, or caffeine; high tobacco consumption (> 10 cigarettes per day); and an inability to complete postural stability assessments due to a fear of falling.

Demographic data, including sex, age, height, weight, body mass index (BMI), geriatric depression scale (GDS), and mini-mental state evaluation (MMSE), were collected from each participant.

### Assessment of postural stability

Each participant was positioned on a posturographic platform from Loran Engineering in Bologna, Italy, to sustain an upright standing posture with arms comfortably at their sides in a quiet environment. In the EO condition, individuals were directed to gaze straight at a fixed point on the wall, positioned 1 m away, for a duration of 30 s. Conversely, in the eyes-closed (EC) condition, subjects were guided to keep their eyes closed for a duration of 30 s. Throughout the tests, alterations in positions and trajectories of the center of pressure (CoP) were computed using specialized software. A series of parameters, including the total track length (TTL; cm), sway area (SA; cm2), mediolateral track length (ML; cm), and anteroposterior track length (AP; cm) were recorded.

### Assessment of risk of falling

To evaluate the risk of falls, we employed the Downton Fall Riks Index (DFRI) (Kasović et al., 2020), a dependable and valid instrument designed to assess five modules: previous falls, education, sensory deficits, mental state, and gait. This leads to 11 distinct risk factors, which are subsequently compiled to generate a score ranging from 0 to 11. A higher score indicated an increased risk of falls. The DFRI is widely utilized as a tool in hospitals, residential communities, and nursing home residents (Vassallo et al., 2004; Del Brutto et al., 2020).

### Circadian rhythm data

Following our established protocol (Zhao et al., 2022), all patients were requested to wear the ActiGraph GT3X + device (ActiGraph, Pensacola, USA) on their non-dominant wrist throughout the day, with exceptions made for swimming or bathing, over the course of 1 week. The ActiLife software (ActiGraph) was utilized to retrieve the data following each wear period. Only data with sufficient wear time were included in the analysis. Actigraphic data were collected at an epoch of 60 s using the corresponding software. Because of the triaxial accelerometer design of the ActiGraph GT3X + device, diurnal vector magnitude (VM) was sampled using the following equation: VM = $\sqrt{X^{2}+Y^{2}+Z^{2}}$ (X, Y and Z represent vector magnitude counts in X-, Y- and Z-axes, respectively).

### Non-parametric analysis

Non-parametric properties were assessed using actigraphic enumeration data, specifically vector magnitude data, to determine the following: interdaily stability (IS), intraday variability (IV), relative amplitude (RA), interdaily coefficient of variation (ICV), time occurrence with associated activity counts for a 10-h period with maximum activity (M10) and a 5-h period with minimum activity (L5) (Van Someren et al., 1999).

IS offers insights into the CR synchronization in response to environmental stimulation with claimed stability. It was determined using the following formula, according to an average 24-h profile (Equation 1):


(1) $$\text{IS}=\frac{n\sum_{h=1}^{p}{\left({\bar{x}}_{h}-\bar{x}\right)}^{2}}{p\sum_{1}^{n}{\left(x_{i}-\bar{x}\right)}^{2}}$$

Based on our previous results, in aged adults, IS ranged from 0.29 to 0.46 in different level of severity among aged patients with small vessel disease, thus an abnormal CR was defined as an IS below 0.30 (Zhao et al., 2023; Zhào et al., 2025).

IV offers data about the fragmentation of the CR within a 24-h profile. It can be determined from hourly raw data using the following equation (Equation 2):


(2) $$IV=\frac{n\sum_{i=2}^{p}{\left(x_{i}-x_{i-1}\right)}^{2}}{\left(n-1\right)\sum_{i=1}^{n}{\left(x_{i}-\bar{x}\right)}^{2}}$$

In the IS and IV formulas, the variables are defined as follows: n and p represent the total number of data points and the daily data entry count, respectively; xh, x and x_i_ represent the mean every hour, the mean data, and raw data hourly, respectively. IS ranges from 0 to 1, with 0 representing Gaussian noise (including normal distribution noise) and 1 representing perfect synchronization. IV ranges from 0 to 2, with a high value indicating a fragmented rhythm.

M10 is the maximum sum for a 10 h continuous activity log, and L5 represents the smallest sum for a 5 h continuous activity log. The ratio of (M10–L5)/(M10 + L5) can be used to calculate the RA (Zhao et al., 2023).

### Sleep quality

The Epworth sleepiness scale (ESS) involves a self-administered questionnaire designed to evaluate an individual’s subjective tendency for daytime sleepiness (Peng et al., 2011).

Sleep variables such as sleep latency (SL), total time in bed (TTB), total sleep time (TST), sleep efficiency (SE), wake after sleep onset (WASO), and times of awakenings (TA) could be automatically analyzed and exported by the ActiLife software.

### Statistical analysis

The differences in demographic and clinical data between the groups were deter-mined using the Student’s *t*-test and chi-square test. A linear regression model, adjusting for sex, age, GDS, and MMSE, was employed to establish the relationship between CR non-parametric variables and postural stability. The linearity and normality were tested and clarified. Normally distributed data were expressed as mean ± standard deviation (SD) or 95% confidence interval (CI). Statistical significance was set at *p* < 0.05. All statistical tests were conducted with SPSS (v25.0; IBM Corp., USA).

## Results

The demographic features of each subject are outlined in Table 1. There were no significant differences in sex, age, weight, height, educational level, GDS, and MMSE s between the HFR and LFR groups. However, HFR subjects demonstrated a remarkably higher DFRI score (2.40 ± 1.15 vs. 1.84 ± 0.55; *p* = 0.036) than LFR individuals. Table 1 also shows the CR information for the two groups. A series of variables reflecting the CR were found to be statistically more disrupted in the HFR group compared to the LFR group. In detail, individuals with a HFR displayed lower IS (0.29 ± 0.22 vs. 0.42 ± 0.19; *p* = 0.040), higher ICV (1.25 ± 0.78 vs. 0.82 ± 0.57; *p* = 0.031), and lower M10 activity counts (80,037 ± 43,492 vs. 111,212 ± 60,094; *p* = 0.043) compared to those in the LFR group. We did not find obvious differences regarding ESS score and sleep quality. Further details are given in Supplementary Table 1.

**Table 1:** Clinical and demographic characteristics of the participants.

Characteristics	HFR (*N*=25)	LFR (*N*=25)	Overall (*N*=50)	*p* value
Men, %	7 (28.00%)	9 (36.00%)	16 (32.00%)	0.544
Age, years	75.60 (5.40)	72.68 (7.66)	74.14 (6.72)	0.126
Height, cm	165.88 (7.49)	167.13 (6.56)	166.50 (6.83)	0.728
Weight, Kg	66.13 (8.60)	74.00 (12.37)	70.06 (11.07)	0.162
Education, years	7.72 (2.69)	7.56 (3.61)	7.64 (3.15)	0.860
BMI, kg/m^2^	24.06 (3.04)	26.47 (3.77)	25.26 (3.54)	0.182
GDS, score	15.16 (6.46)	14.80 (7.15)	14.98 (6.74)	0.853
MMSE, score	25.60 (5.16)	24.88 (3.23)	25.24 (4.28)	0.557
DFRI, score	2.40 (1.15)	1.84 (0.55)	2.12 (0.94)	0.036*
IS	0.29 (0.22)	0.42 (0.19)	0.35 (0.21)	0.040*
IV	0.85 (0.38)	0.95 (0.35)	0.90 (0.37)	0.337
ICV	1.25 (0.78)	0.82 (0.57)	1.05 (0.71)	0.040*
M10, activity counts	80037 (43492)	111212 (60094)	95624 (54252)	0.041*
L5, activity counts	5450 (4050)	5540 (3250)	5495 (3571)	0.930
ESS, score	2.61 (2.48)	2.67 (3.51)	2.64 (3.02)	0.948

Mean (Standard Deviation) for Age, Height, Weight, BMI, GDS, MMSE, DFRI, IS, IV, M10, L5 and RA.

Number (Percentage) for Gender.

*p<0.05 HFR relative to LFR.

BMI, Body Mass Index; GDS, Geriatric Depression Scale; DFRI, Downton Fall Risk Index; MMSE, Mini-mental State Evaluation; IS, interdaily stability; IV, intradaily variability; M10, activity counts during 10 h period with highest activity; L5, activity counts during 5 h period with lowest activity; ESS, Epworth Sleepiness Scale.

Additionally, we explored the relationship between CR disruption and increased postural sway using linear regression analysis. Under the EO condition, we did not observe an obvious association between CR variables (IS, IV, M10, L5) and postural variables, such as TTL, SA, ML, and AP. However, under the EC condition, L5 was positively associated with TTL (*p* = 0.026, standardized *β* = 0.841), SA (*p* = 0.007, standardized *β* = 1.050), and ML (*p* < 0.001, standardized *β* = 1.267). After adjusting for age, sex, GDS, and MMSE, the association was still significant between SA (*p* = 0.016, standardized *β* = 0.887), and ML (*p* < 0.001, standardized *β* = 1.230). Further details are provided in Tables 2, 3, as well as Supplementary Figure 1. We did not find significant association between sleep quality and postural sway. Details are listed in Supplementary Tables 2, 3.

**Table 2:** Linear regression for association between CR variables and postural control under EO condition.

CR variables	Postural control variables														
	TTL			AV			SA			ML			AP		
	*β*	95% CI	*p*	*β*	95% CI	*p*	*β*	95% CI	*p*	*β*	95% CI	*p*	*β*	95% CI	*p*
IS															
Model 1	0.099	−3540.25~5108.10	0.717	0.100	−58.97~85.18	0.716	−0.290	−1662.98~533.93	0.306	−0.191	−14.50~7.19	0.501	0.060	−5.64~7.01	0.828
Model 2	−0.078	−5547.62~4317.99	0.803	−0.780	−99.44~147.54	0.803	−0.277	−1763.15638.86	0.378	−0.282	−17.66~6.86	0.379	0.133	−6.06~8.65	0.724
IV															
Model 1	−0.071	−2484.42~1833.87	0.763	−0.072	−41.45~30.53	0.761	−0.011	−560.53~536.42	0.965	−0.015	−5.54~5.25	0.950	0.005	−3.12~3.19	0.982
Model 2	−0.023	−2381.15~2166.84	0.925	−0.024	−39.73~36.08	0.923	−0.018	−583.87~544.19	0.944	0.013	−5.61~5.69	0.990	0.001	−3.39~3.39	0.999
M10															
Model 1	0.156	−0.008~−0.018	0.461	0.156	0.000~0.000	0.461	−0.232	−0.005~0.002	0.289	−0.144	0.000~0.000	0.513	0.135	0.000~0.000	0.529
Model 2	0.008	−0.015~0.016	0.974	0.008	0.000~0.000	0.975	−0.212	−0.005~0.002	0.395	−0.216	0.000~0.000	0.394	0.192	0.000~0.000	0.452
L5															
Model 1	0.337	−0.013~0.340	0.100	0.337	−0.001~0.005	0.140	0.055	−0.031~0.043	0.732	0.143	0.000~0.001	0.374	0.298	0.000~0.000	0.062
Model 2	0.274	−0.039~0.297	0.130	0.274	−0.001~0.005	0.130	−0.555	−0.038~0.045	0.864	0.087	0.000~0.001	0.636	0.328	0.000~0.000	0.079

Model 1 represents the association between CR variables and postural control without adjustment. Model 2 represents the association between CR variables and posture control adjusted for age, gender, GDS and MMSE.

*p<0.05.

IS, Interdaily Stability; IV, Intradaily Variability; M10, 10 h period with the highest activity; L5, 5 h period with the lowest activity; TTL, Total Track Length; AV, Average Velocity; SA, Sway Area; ML, Mediolateral track length; AP, Anteroposterior track length; GDS, Geriatric Depression Scale; MMSE, Mini-mental State Evaluation; CI, confidence intervals.

**Table 3:** Linear regression for association between CR variables and postural control under EC condition.

CR variables	Postural control variables														
	TTL			AV			SA			ML			AP		
	*β*	95% CI	*p*	*β*	95% CI	*p*	*β*	95% CI	*p*	*β*	95% CI	*p*	*β*	95% CI	*p*
IS															
Model 1	−0.228	−900.11~359.36	0.392	−0.227	−14.99~6.00	0.393	−0.205	−1858.45~811.19	0.434	−0.411	−15.52~0.36	0.061	0.060	−9.11~4.71	0.828
Model 2	−0.059	−747.72~607.95	0.836	−0.058	−12.44~10.16	0.839	−0.082	−1685.19~1256.79	0.776	−0.358	−15.53~2.31	0.142	0.133	−5.65~7.76	0.724
IV															
Model 1	0.124	−229.06~607.95	0.587	0.123	−3.83~6.65	0.590	0.062	−575.08~757.92	0.784	0.028	−3.67~4.26	0.881	0.005	−3.07~3.83	0.982
Model 2	0.107	−238.78~386.18	0.636	0.105	−4.00~6.42	0.642	0.076	−568.13~792.26	0.741	0.065	−3.42~4.81	0.736	0.001	−2.73~3.45	0.999
M10															
Model 1	0.200	−0.001~0.003	0.330	0.200	0.000~0.000	0.331	0.077	−0.003~0.005	0.701	−0.033	0.000~0.000	0.842	0.135	0.000~0.000	0.529
Model 2	0.376	0.000~0.004	0.101	0.375	0.000~0.001	0.101	0.217	−0.002~0.007	0.344	0.062	0.000~0.000	0.745	0.192	0.000~0.000	0.452
L5															
Model 1	0.375	−0.013~0.340	0.015*	0.376	0.000~0.001	0.015*	0.449	0.023~0.113	0.004**	0.682	0.000~0.001	0.000***	0.298	0.000~0.000	0.062
Model 2	0.428	0.005~0.005	0.012*	0.429	0.000~0.001	0.011*	0.490	0.024~0.125	0.005**	0.781	0.001~0.001	0.000***	0.328	0.000~0.000	0.079

Model 1 represents the association between postural control and CR variables without adjustment. Model 2 represents the association between CR variables and posture control adjusted for age, gender, GDS and MMSE.

*p<0.05, **p<0.001.

IS, Interdaily Stability; IV, Intradaily Variability; M10, 10 h period with the highest activity; L5, 5 h period with the lowest activity; TTL, Total Track Length; AV, Average Velocity; SA, Sway Area; ML, Mediolateral track length; AP, Anteroposterior track length; GDS, Geriatric Depression Scale; MMSE, Mini-mental State Evaluation; CI, confidence intervals.

## Discussion

Our current investigation observed an abnormal CR pattern, characterized by decrements in CR stability and lower M10 activity counts, in elderly individuals with a high fall risk. Notably, the results indicated a correlation between increased postural sway. and compromised CR performance, specifically regarding L5 activity counts, under the EC condition, as opposed to EO condition.

Although previous research (Umemura et al., 2022) has suggested the potential impact of CR disruption on posture control, definitive conclusions have been elusive. In a study of 30 patients with chronic sleep issues, Furtado et al. (2016) found that individuals with higher L5 activity counts exhibited poorer performance in postural tests compared to those with lower L5 activity counts. Building on our preliminary findings, it became apparent that aged adults with impaired postural control displayed lower M10 activity counts. It is worth noting that L5 denotes “resting levels during the night,” with a higher L5 indicating less restful sleep. In contrast, M10 represents “how active the wake periods are” (Mitchell et al., 2017). Our current results, in conjunction with Furtado et al. (2016) observations, suggest a plausible link between CR instability and its impact on posture control. Furthermore, both the IS and ICV differed between groups, implying that CR instability might be another factor related to postural abnormalities in aged individuals. As has been revealed by Bashir et al. (2020), night shifts were associated with a high prevalence of dizziness symptoms (Benign Paroxysmal Positional Vertigo). A potential explanation was that disturbance of the normal CR commonly found in night shift doctors and nurses could induce poor quality sleep and vestibular system stress. The existing evidence indicates that CR disturbances are associated with inflammatory markers (Parsons et al., 2017). This leads us to infer that neuroinflammation induced by CR disruption may be the underlying postural instability mechanism. There are alternative explanations, particularly the possibility of reverse causality reducing activity levels and thereby altering actigraphy-derived CR measures. A bidirectional link between CR disorders and frailty has been proposed (Maekawa and Kume, 2019; Pan et al., 2023). Considering that muscle strength is one of the major domains for multifactorial falls risk assessment (Montero-Odasso et al., 2022), we assumed that frailty might be potential pathway between CR abnormalities and increase postural sway.

In addition, the relationships between CR variables and postural stability varied notably under different conditions. No evident association was observed between CR variables and postural variables under the EO condition; however, a clear relationship emerged under the EC condition. It is worth noting that changes in postural variables following in the EC condition are believed to underscore the significance of visual input in postural control (Robillard et al., 2011). Sleep deprivation studies reported that experimental sleep loss could deteriorate the useful visual field (Rogé et al., 2003). These results suggest that older individuals lean heavily on visual information to stabilize their postural control. In other words, older adults with CR disruption may face an increased risk of falling, especially in dark or complex conditions (e.g., a room with excessive furniture or an unfamiliar environment like a hotel or hospital room). Accordingly, improvements in the indoor light system could be a potential way to meet the demand from both visual and non-visual aspects (Brown et al., 2022). Suprapostural visual tasks are interesting and useful, and may be a common feature of everyday life (Stoffregen, 2016). It has been evidenced that postural control can be influenced by variations in oculomotor demand (Stoffregen et al., 2007). Thus, more complex future studies could elucidate how the CR may be related to the pervasive modulation of postural kinematics in relation to “suprapostural” visual tasks.

Regarding the directionality of the association, we observed that L5 was linked to track length in the ML direction rather than the AP direction under the EC condition. Previous studies indicated that sleep deprivation predominantly influenced body sway in the AP direction, not the ML direction (Robillard et al., 2011; Umemura et al., 2019). These disparities might stem from distinct underlying pathobiological changes induced by sleep loss and CR disruption. Our study did not observe significant differences in the ESS values, highlighting the need for a more in-depth examination of sleep parameters to distinguish between sleep disorders and CR disruption.

Additionally, previous research has established a subtle daytime effect on postural stability in older individuals, which is primarily present in the afternoon (Rym et al., 2019). In the current study, postural stability tests for each participant were conducted at random time points in the afternoon; however, it is noteworthy that in sleep deprivation procedures, participants are typically instructed to perform balance tests in the morning (Umemura et al., 2019).

Nevertheless, this research has some limitations. First, the sample size was considerably small, without correction applied for multiple comparisons, further studies with a larger sample are needed to confirm the findings. Second, more CR variables, especially those derived from parametric methods such as the extended cosine-based method (Van Someren et al., 1999), are needed to illustrate CR patterns. Third, we did not clarify the visually demanding instruction of the participants during the EO task. Forth, other clinical risk factor (e.g., fall history, frailty markers) should be collected and analyzed in future studies as covariates.

In summary, our preliminary findings affirm that the risk of falls increases with alterations in chronobiology. An increase in body sway is linked to CR disruption in older adults under the EC condition. There should be a heightened focus on enhancing the environmental light system to mitigate the elevated risk of falls in older individuals with CR abnormalities.

## Data Availability

The original contributions presented in the study are included in the article/Supplementary material, further inquiries can be directed to the corresponding author.

## Supplementary material

SUPPLEMENTARY FIGURE 1 Scatter graphs demonstrating the relationship between L5 and SA, as well as ML. SA, sway area; ML, mediolateral track length.
