# Supplementary figures and images for: Circadian disruption is associated with altered postural control in aged individuals under eye closed condition
Source: Front Neurosci. 2025 Apr 30;19:1574544. doi: 10.3389/fnins.2025.1574544 (PMC12076166; doi:10.3389/fnins.2025.1574544)

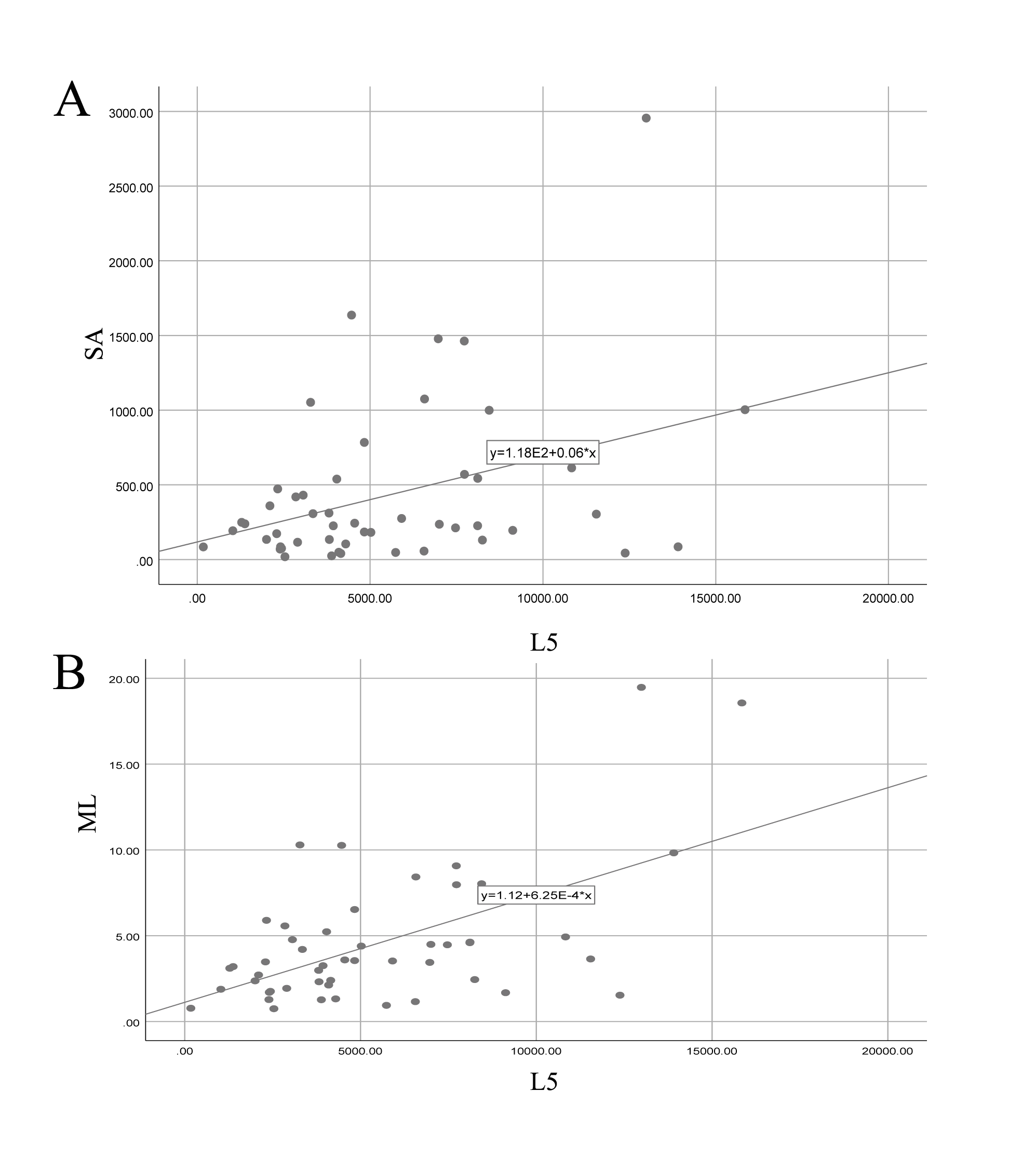

Supplement: SUPPLEMENTARY FIGURE 1 — Scatter graphs demonstrating the relationship between L5 and SA, as well as ML. SA, sway area; ML, mediolateral track length. [file Image_1.tif]
