# Supplementary material for: Circadian disruption is associated with altered postural control in aged individuals under eye closed condition
Source: Front Neurosci. 2025 Apr 30;19:1574544. doi: 10.3389/fnins.2025.1574544 (PMC12076166; doi:10.3389/fnins.2025.1574544)
Supplement: Supplementary file 2 [file Table_1.docx]

**Supplementary Table 1 Subjective tendency for daytime sleepiness and objective sleep quality of the participants.**

| Characteristics | HFR  (N=25) | LFR  (N=25) | Overall  (N=50) | P value | |
| --- | --- | --- | --- | --- | --- |
| ESS, score  SL, min  TTB, min | 2.61(2.48)  25.53(15.10)  445.19(38.18) | 2.67(3.51)  18.38(13.43)  439.53(28.88) | 2.64(3.02)  21.95(14.59)  442.36(33.61) | | 0.948  0.090  0.565 |
| TST, min | 362.08(44.55) | 358.27(51.90) | 360.24(47.89) | | 0.799 |
| SE, %  WASO, min  TA, times | 83.38(6.59)  50.85(30.63)  19.88(10.47) | 83.07(10.58)  53.02(40.26)  17.63(9.06) | 82.72(8.72)  51.94(35.41)  18.75(9.76) | | 0.788  0.834  0.430 |

Mean (Standard Deviation) for ESS, SL, TTB, TST, SE, WASO, TA.

ESS: The Epworth sleepiness scale, SL: Sleep Latency, TTB: Total Time in Bed, TST: Total Sleep Time, SE: Sleep Efficiency, WASO: Wake After Sleep Onset.
